# Preface to the Special Issue: Quantum Probability and Randomness V

**DOI:** 10.3390/e27101010

**Published:** 2025-09-26

**Authors:** Andrei Khrennikov, Karl Svozil

**Affiliations:** 1 Center for Mathematical Modeling in Physics and Cognitive Sciences, Linnaeus University, SE-351 95 Växjö, Sweden; 2 Department of Physics, Technical University of Vienna, 1040 Vienna, Austria; karl.svozil@tuwien.ac.at

## Preface

In recent years, the interface between probability theory, randomness, and quantum foundations has witnessed rapid developments, both in experimental and theoretical domains. This fifth Special Issue of “Quantum Probability and Randomness” gathers twenty contributions that deepen our understanding of quantum probability’s formal structure and its diverse applications—from randomness certification and contextuality to cognition and cosmology.

At the same time, this Special Issue reflects the ongoing dialogue in the quantum probability and foundations community, capturing both longstanding and emerging perspectives. For decades, much of the discourse has centered on the distinction between vector spaces and power sets, emphasizing how the “vector world” of quantum theory differs from classical physics. While this line of inquiry has been foundational, it can sometimes appear repetitive or narrowly focused.

At the same time, a more challenging and innovative line of research investigates the extent to which phenomena traditionally considered uniquely quantum—such as contextuality, intrinsic undecidables, and non-classical correlations—can manifest in classical systems. Contributions in this issue explore these subtler intersections, including automaton partition logics, Gödelian-type intrinsic undecidables, and other classical analogs of quantum features.

The Växjö annual conference series (started in 2000) have historically sought to accommodate both perspectives: the careful formal analysis of quantum–classical distinctions and the exploration of surprising “quantum-like” features in classical contexts. This dual spirit is reflected throughout the proceedings, offering readers a rich and diverse set of conceptual approaches that advance our understanding of both quantum theory and its classical analogs.

The collection begins with works probing how quantum computers—and especially noisy, real hardware—handle entanglement, fidelity, and entropy in forming GHZ states [contribution 1], highlighting the practical trade-offs in the current era. Several contributions explore “quantum-like” behaviors in cognition and perception, such as contextuality in human judgments of real vs. AI-generated images [contribution 2], which suggest that non-classical probabilistic models may be necessary outside physics proper.

Foundational issues such as measurement theory are revisited in new light: negative-result experiments are reinterpreted without invoking ad hoc collapse postulates [contribution 16]; conditional values of observables are analyzed via local decompositions that partition amplitude vs. phase contributions [contribution 17]. Hypergraph and chromatic criteria for contextuality are developed to sharpen the distinction between classical and quantum bounds, particularly in high-dimensional settings [contributions 9,11].

Other contributions expand the formal landscape by integrating group-algebraic methods [contribution 20], generalized probability models [contribution 12], and stochastic motion formulations of quantum mechanics [contribution 5,14]—suggesting that alternative frameworks may illuminate under-explained aspects of quantum behavior. Applications are equally diverse, from randomness expansion protocols using parity-oblivious codes [contribution 6] to statistical testing of real random number generators [contribution 13] and theoretical treatments of quantum vacuum effects in cosmology [contribution 14].

Now we briefly summarize the content of the contributions to this Special Issue:

1. Entropy, Fidelity, and Entanglement During Digitized Adiabatic Quantum Computing to Form a Greenberger–Horne–Zeilinger (GHZ) State [contribution 1]
  This study investigates the generation of a three-qubit GHZ state using digitized adiabatic quantum computing. The authors analyze the dynamics of entropy, fidelity, and entanglement throughout the adiabatic process, providing insights into the efficiency and reliability of this quantum computing approach.
2. Are Human Judgments of Real and Fake Faces Quantum-like Contextual? [contribution 2]
  This paper explores the hypothesis that human judgments of real versus fake faces exhibit quantum-like contextuality. Through experimental data and analysis, the authors demonstrate that these judgments cannot be fully explained by classical probability theory, suggesting the need for quantum-inspired models in cognitive science.
3. Bohmian Chaos and Entanglement in a Two-Qubit System [contribution 3]
  The authors examine the interplay between Bohmian mechanics and quantum entanglement in a two-qubit system. They identify critical points in the Bohmian flow and analyze their relationship with the onset of chaos, providing a deeper understanding of quantum dynamics in entangled systems.
4. A Missing Link: The Double-Slit Experiment and Quantum Entanglement [contribution 4]
  This article reinterprets the double-slit experiment by establishing a novel connection between the experiment and quantum entanglement. The author argues that this perspective offers new insights into the fundamental nature of quantum phenomena and challenges traditional interpretations.
5. Motion of Quantum Particles in Terms of Probabilities of Paths [contribution 5]
  The paper presents a path integral approach to describing the motion of quantum particles. By analyzing the probabilities of various paths, the author provides a comprehensive framework for understanding quantum trajectories and their implications for quantum mechanics.
6. Semi-Device-Independent Randomness Expansion Using Parity-Oblivious Quantum Random Access Codes [contribution 6]
  This work introduces a semi-device-independent randomness expansion protocol utilizing parity-oblivious quantum random access codes. The authors demonstrate how true quantum randomness can be certified through the violation of a two-dimensional quantum witness, enhancing the robustness of randomness generation.
7. Intrinsic and Measured Information in Separable Quantum Processes [contribution 7]
  The authors explore the information-theoretic properties of separable quantum processes, focusing on intrinsic and measured information. They provide methods for approximating information sources with independent and identically distributed models, offering insights into the structure of quantum stochastic processes.
8. A Note on the Relativistic Transformation Properties of Quantum Stochastic Calculus [contribution 8]
  This paper discusses the relativistic transformation properties of quantum stochastic calculus. The author derives the transformation rules between inertial observers and examines the implications for quantum open system dynamics in relativistic contexts.
9. Chromatic Quantum Contextuality [contribution 9]
  The study introduces the concept of chromatic quantum contextuality, extending the framework of quantum contextuality to include color-based structures. The author explores the implications of this extension for understanding the non-locality and non-contextuality in quantum systems.
10. Fitting Copulas with Maximal Entropy [contribution 10]
  This article addresses the problem of fitting copulas with maximal entropy. The author presents methods for constructing copulas that maximize entropy, providing tools for modeling dependencies in multivariate distributions within the context of quantum information theory.
11. Quantum Contextual Hypergraphs, Operators, Inequalities, and Applications in Higher Dimensions [contribution 11]
  The paper extends the concept of quantum contextuality to higher dimensions using hypergraphs. The author develops operator inequalities and explores their applications, offering a geometric perspective on quantum contextuality and its implications for quantum mechanics.
12. Problem of Existence of Joint Distribution on Quantum Logic [contribution 12]
  This study investigates the problem of the existence of a joint distribution on quantum logic. The author examines the conditions under which such distributions can exist, contributing to the understanding of the foundations of quantum probability theory.
13. Statistical Testing of Random Number Generators and Their Improvement Using Randomness Extraction [contribution 13]
  The authors discuss statistical methods for testing random number generators and propose improvements using randomness extraction techniques. They highlight the importance of these methods in ensuring the reliability and security of quantum random number generation.
14. Effects of the Quantum Vacuum at a Cosmic Scale and of Dark Energy [contribution 14]
  This paper explores the effects of the quantum vacuum on a cosmic scale, particularly in relation to dark energy. The author examines how quantum fluctuations contribute to the accelerated expansion of the universe, offering insights into the interplay between quantum mechanics and cosmology.
15. Statistical Properties of Superpositions of Coherent Phase States with Opposite Arguments [contribution 15]
  The study analyzes the statistical properties of superpositions of coherent phase states with opposite arguments. The author investigates the implications for quantum coherence and interference, providing a deeper understanding of quantum state superpositions.
16. On the Negative Result Experiments in Quantum Mechanics [contribution 16]
  This article examines negative result experiments in quantum mechanics, focusing on their role in testing the foundations of quantum theory. The author discusses the significance of these experiments in challenging and refining quantum mechanical models.
17. Conditional Values in Quantum Mechanics [contribution 17]
  The paper introduces the concept of conditional values in quantum mechanics, extending the framework of quantum measurements. The author explores how these values can be used to model conditional probabilities and their implications for quantum state determination.
18. Statistical Signatures of Quantum Contextuality [contribution 18]
  This study identifies statistical signatures of quantum contextuality, providing experimental criteria for detecting contextual effects in quantum systems. The author discusses the significance of these signatures in understanding the non-classical nature of quantum mechanics.
19. Correlations in the EPR State Observables [contribution 19]
  The authors investigate the correlations in the EPR state observables, focusing on the violation of Bell inequalities. They analyze the implications for quantum entanglement and non-locality, contributing to the foundational understanding of quantum correlations.
20. The Group-Algebraic Formalism of Quantum Probability and Its Applications in Quantum Statistical Mechanics [contribution 20]
  This paper presents a group-algebraic formalism of quantum probability, applying it to quantum statistical mechanics. The author develops a framework for understanding quantum probabilities in terms of group theory, offering new perspectives on quantum statistical systems.

Altogether, the papers in this issue reflect a dual thrust: first, to reinforce the mathematical and conceptual foundations of quantum probability and its randomness; and second, to broaden its reach into applications in computation, cognition, and cosmology. We believe these works collectively help to clarify the boundaries between classical and quantum uncertainty, challenge prevailing assumptions, and open new pathways for research.

We hope that readers will find here not only deep theoretical insights but also inspiration for future explorations—whether in proving novel inequalities, constructing more robust random protocols, or discovering quantum-like phenomena beyond the traditional quantum domain.

